# Prevalence and Genotyping of High Risk Human Papillomavirus in Cervical Cancer Samples from Punjab, Pakistan

**DOI:** 10.3390/v6072762

**Published:** 2014-07-17

**Authors:** Abida Siddiqa, Maidah Zainab, Ishtiaq Qadri, Muhammad Faraz Bhatti, Joanna L. Parish

**Affiliations:** 1 Atta-ur-Rahman School of Applied Biosciences (ASAB), National University of Sciences and Technology (NUST), Sector H-12, Kashmir Highway, Islamabad 44000, Pakistan; E-Mails: abida.10.tfrphd.67@asab.nust.edu.pk (A.S.); maidahzainab@gmail.com (M.Z.); 2 National Center of Virology and Immunology, Islamabad 44000, Pakistan; E-Mail: Ishtiaq80262@yahoo.com; 3 School of Cancer Sciences, Cancer Research UK Birmingham Cancer Centre, College of Medical and Dental Sciences, University of Birmingham, Birmingham B15 2TT, UK

**Keywords:** HPV, prevalence, Pakistan, cervical cancer

## Abstract

Cervical cancer is the third most common cause of cancer-related death in women worldwide. Infection with high-risk human papillomavirus (HPV) is established as the cause of cervical carcinoma, therefore, high risk HPV detection may have prognostic significance for the women who are at increased risk of disease progression. The paucity of data on the incidence of cervical cancer in Pakistan makes it difficult to determine disease burden. Even less information is available regarding the prevalent HPV strains in cervical specimens collected from this region. Cervical cancer is a neglected disease in Pakistan in terms of screening, prevention, and vaccination. Identification and accurate genotyping of the virus burden in cancer specimens is important to inform intervention policies for future management of HPV associated disease and to potentially stratify patients dependent on HPV status. In this study, detection and genotyping of HPV types 16 and 18 from 77 cervical specimens were carried out. Consensus primers GP5+/GP6+, which detect 44 genital HPV types, and type specific primers (TS16 and TS18) were used in conjunction with newly designed type specific primers. Using a combination of these methods of detection, a total of 94.81% (95% CI ±4.95) of cervical lesions were positive for HPV. Single infections of HPV16 were detected in 24.68% (95% CI ±9.63) of total samples and HPV18 was found in 25.97% (95% CI ±9.79) samples. Interestingly, a high proportion of samples (40.26%, 95% CI ±10.95) was positive for both HPV16 and 18, indicating a higher incidence of co-infection than previously reported for similar ethnic regions. The HPV genotype of 3.90% of HPV positive samples remained undetected, although these samples were positive with the GP5+/GP6+ primer set indicating infection with an HPV type other than 16 or 18. These data indicate that the overall incidence of high risk HPV infection in cervical cancer and intraepithelial neoplasia specimens in Punjab, Pakistan is in line with the worldwide prevalence, but that the incidence of HPV16 and 18 co-infections in our cohort is higher than that previously reported.

## 1. Introduction

Cervical cancer is recognized as the third most common type of cancer in women worldwide and the second most prevalent cancer type and cause of cancer-related mortality in women in developing countries [1]. High-risk human papillomavirus (HPV) infection has been established as the main cause of cervical cancer [2]. Over 200 types of HPV are predicted to exist [3], including 30 types that are sexually transmitted and result in cervical infections [4]. Based on the frequency of detection in cervical cancer, HPV genotypes are sub-divided into high-risk HPV types (16, 18, 31 and 45), intermediate-risk types (33, 35, 39, 51, 52, 56, 58, 59, and 68) and low-risk types (6, 11, 42 and 44) [5]. 

Most of the HPV-induced changes in cervical cells are transient and about 90% of them regress spontaneously within 12 to 36 months as the immune system eliminates the virus [4]. Papanicolaou (Pap) smear screening has played a significant role in the diagnosis of pre-malignant and malignant lesions in the cervix and has decreased the associated mortality rate in countries that have a robust screening program in place, however such screening programs in developing countries are limited or non-existent [1]. Since persistent infection with high-risk HPV types can result in cervical intraepithelial neoplasia (CIN) of different grades and invasive cancer within several years [6], and progression to cancer can be prevented by early detection of abnormalities and subsequent treatment, it is important to establish cost effective, sensitive, and accurate cervical screening protocols within routine clinical practice [7]. 

Cervical cancer is the fourth most common cancer in Pakistan with an age standardized risk (ASR) per 100,000 women of 7.5, although these data come from a limited sampling of the population of Pakistan (~1%) and the incidence of cervical cancer is likely to be on the rise [8,9]. These data indicate that while the cervical cancer burden in Pakistan is low compared to the worldwide ASR of 15.2 per 100,000 women, in comparison to the risk within the Extended Middle East Region (ASR 3.4 per 100,000 women), the cervical cancer burden is likely to be high [9]. The reasons for this are unclear, and further work is required to determine an accurate assessment of the cervical cancer burden and the HPV types associated with cancer development in Pakistan. Indeed, the role of HPV in cervical cancer in the Pakistani population has not been broadly studied. The only epidemiological data that has been reported was collected from patients from Karachi, Sindh [10,11,12]. However, these studies report conflicting results with HPV detected in 18%, 98.3%, and 92.2% of cervical cancer specimens, respectively [10,11,12]. Hence, there is a lack of consistent data on the prevalent HPV genotypes in Karachi and no available data on HPV prevalence in cervical cancer and neoplasia specimens in Punjab, Pakistan. While the incidence of cervical cancer in developed countries is declining, consistent with the introduction of rigorous cervical screening programs, successful treatment strategies, and vaccination practices, such interventions have yet to be implemented in Pakistan. 

Organized biobanking in Pakistan is a very reasonable concept in the pathology of the cervix as slides and formalin fixed paraffin embedded blocks are routinely stored for the period of 5 to 10 years for a number of reasons [13]. Polymerase chain reaction (PCR) is an efficient tool for the detection of different pathogens such as HPV in these stored samples [14]. The present study was conducted using paraffin embedded and retrospective cervical cancer tissues of patients from different areas of Punjab, Pakistan in order to determine the prevalence of HPV infection in cervical cancer specimens and the relevant HPV genotypes associated with disease. 

## 2. Materials and Methods

### 2.1. Sample Collection

A total of 74 formalin-fixed paraffin-embedded samples of cervical cancer tissue biopsies was collected from major hospitals in Punjab, Pakistan; Shifa International Hospital (SIH), and Pakistan Institute of Medical Science (PIMS), Islamabad (n = 13), Nishter Hospital (NH), Multan (n = 16), Allied Hospital (AH), Faisalabad (n = 11), Mayo Hospital (MaH), Lahore (n = 21), Holy Family Hospital (HFH) and Military Hospital (MH), Rawalpindi (n = 16). The samples were taken from patients aged 25–70 years (with mean age 46 and median age 50, where ages were known) who were treated during the period of January 2007–June 2010. Consent of the patient for the use of their samples for research purpose was taken (reviewed by NUST, Islamabad, Pakistan).

Fresh samples were obtained from patients attending the Military Hospital (MH), Rawalpindi. All samples (two uterine cervix biopsies and one hysterectomy) were diagnosed for cervical cancer by the resident consultant histopathologist of the institute. The cervix was excised as part of their treatment and stored in phosphate buffer saline (137 mM NaCl, 2.7 mM KCl, 10 mM Na_2_HPO_4_, 2 mM KH_2_PO_4_, pH 7.4) immediately following removal.

### 2.2. DNA Extraction from Formalin Fixed Paraffin Embedded (FFPE) and Fresh Tissue Samples

Prior to sectioning, tissue blocks were placed at −20 °C for 1 hour. Sections of 5–20 μm were cut from tissue biopsy samples on a microtome (CUT 6062, SLEE Mainz) using disposable blades and placed in 1.5 mL Eppendorf tubes. To avoid contamination of samples, blades were changed after every 10th sample and the microtome and blade were thoroughly cleaned with distilled water and ethanol between each specimen. Ten to twenty sections of each sample were taken and only the four to six sections were processed for PCR. In addition, an HPV negative tissue sample (atrophic endometrium) was sectioned between each of the cervical specimens. DNA was then extracted from the negative control samples alongside the cervical samples and amplified alongside the experimental samples with primer sets GP5+/6+, C16E7/C18E7, TS16/TS18. It was ensured that the PCR reactions in the negative control samples were negative in all cases included in our analysis. 

The sections were deparaffinized in 1 mL of xylene and shaken vigorously for 2 min followed by centrifugation at 12,200× *g* for 10 min. The supernatant was removed and the pellets were then washed again in xylene to avoid carryover of residual paraffin. The pelleted tissue was treated with 1 mL of 100% (w/v) ethanol followed by centrifugation (16,200× *g* for 10 min) so as to remove residual xylene from the tissue. The supernatant was discarded and the tissue washed with 1 mL of 70% (w/v) ethanol before re-centrifugation at 16,200× *g* for 10 min. The supernatant was discarded and the tissue pellet air-dried. Following homogenization, the dry pellet was suspended in 400 μL digestion buffer (50 mM Tris-HCl, pH 8.5, 1 mM EDTA, and 0.5% Tween 20) and 0.25 mg/mL Proteinase K added (Invitrogen, Darmstadt, Germany). The mixture was incubated at 56 °C overnight. Following digestion, the samples were heated to 95 °C for 10 min and briefly centrifuged. The supernatant containing the DNA was transferred to a new 1.5 mL Eppendorf tube and stored at −20 °C for future use. 

Fresh biopsies (1 mm sections) were directly homogenized in 400 μL of digestion buffer followed by proteinase K treatment as mentioned above, prior to the storage of extracted DNA at −20 °C.

### 2.3. DNA Extraction from HeLa Cells

HeLa belong to an HPV18 positive cervical carcinoma cell line and were cultured in Dulbecco’s Modified Eagle Medium (DMEM) plus 10% fetal bovine serum. Cells were harvested by scraping and transferred to a 1.5 mL Eppendorf tube. The tube was centrifuged for 5 min at 12,200× *g*. The pellet was then resuspended in 200 μL of PBS (pH 7.4) and 2 mg/mL proteinase K was added. DNA was then isolated using QIAamp^®^ DNA Mini Kit (Qiagen). All primer sets used in this study were tested with DNA extracted from HeLa cells to ensure that HPV16-specific primer sets were negative and the HPV18 primer sets were positive. 

### 2.4. Polymerase Chain Reaction

DNA from each sample was amplified by PCR with the primer sets described in Table 1. A 20 μL reaction was assembled that contained 100–300 ng sample DNA, 1x *Taq* polymerase buffer (Fermentas), 0.1 mM deoxynucleotide triphosphate (dNTPs), 2.5 mM MgCl_2_, 1U of *Taq* polymerase (Fermentas), and 0.5 pmol of each primer. The PCR conditions for each primer set are detailed in Table 2. DNA from each sample was extracted in duplicate and each PCR reaction was performed in duplicate for each independent extraction to ensure consistency of the results. PCR reactions were carried out individually for each primer set and the bands produced in five reactions with each primer set were sequenced to ensure specificity of amplification. 

The amplified products were analyzed on a 1.5% agarose gel, stained with ethidium bromide and visualized on a UV transilluminator. Beta-globin primers were used to check the quality of the DNA. The amplification was carried out in the presence of negative and positive controls; DNA from cervical cancer tissue samples positive for HPV (confirmed by sequencing) was used as a positive control for HPV16 and a negative control for HPV18-specific primer sets and DNA from HeLa cell line was used as positive control for HPV18 and a negative control for HPV16-specific primer sets.

**Table 1:** Primers used for detection and genotyping of human papillomavirus (HPV) 16 and 18.

Primer	Sequence (5'-3')	Target	Product Size (bp)	Reference
PC03	ACACAACTGTGTTCACTAGC	β-globin	110	[15]
PC04	CAACTTCATCCACGTTCACC			
GP5+	TTTGTTACTGTGGTAGATACTAC	L1	150	[16]
GP6+	GAAAAATAAACTGTAAATCATATTC			
New TS 16	GGTCGGTGGACCGGTCGATG	E6 HPV 16	96	[17]
	GCAATGTAGGTGTATCTCCA			
New TS 18	CCTTGGACGTAAATTTTTGG CACGCACACGCTTGGCAGGT	L1 HPV 18	115	[17]
C16E7F	GGGGAATTCGCATGGAGATACACCTACATTC	E7 HPV 16	297	Current study
C16E7R	GGGCTCGAGTGGTTTCTGAGAACAGATGG			
C18E7F	GGATCCGCATGGACCTAAGGCAACATT	E7 HPV 18	318	Current study
C18E7R	GAATTCGCTGCTGGGATGCACACCA			

**Table 2:** Polymerase chain reaction (PCR) conditions for individual primer sets.

Primer Name	Hot Start	Denaturation	Annealing	Extension	Final Extension
		35 cycles			
PC03/PC04	94 °C, 4 min	94 °C, 30 s	54 °C, 45 s	72 °C, 30 s	7 min, 72 °C
GP5+/GP6+	94 °C, 4 min	94 °C, 30 s	45 °C, 45 s	72 °C, 30 s	7 min, 72 °C
New TS 16	94 °C, 4 min	94 °C, 45 s	58 °C, 45 s	72 °C, 30 s	7 min, 72 °C
New TS 18					
C16E7					
C18E7					

## 3. Results and Discussion

### 3.1. HPV Status of Cervical Cancer Biopsies

Cervical cancer tissue biopsies and cervical intraepithelial lesions of different degrees were obtained from five different hospitals in Punjab as described in the materials and methods section. Tissue from patients with cervical lesions was obtained regardless of ethnic, social or economic background. A total of 77 cervical cancer biopsies were histopathologically identified as squamous cell carcinoma (SCC; n = 43), adenocarcinoma (AC; n = 1), adenosquamous carcinoma (ASC; n = 3), high grade squamous intraepithelial lesion (HSIL; n = 11), low grade squamous intraepithelial lesion (LSIL; n = 4) and chronic cervicitis/metaplasia (n = 15) (Table 3). The histopathological distribution of our cervical cancer specimens is consistent with that previously reported in Karachi South, Pakistan [18].

**Table 3:** Histopathological classification and HPV status of cervical cancer biopsies SCC; Squamous cell carcinoma, AC; Adenocarcinoma, ASC; Adenosquamous carcinoma, HSIL; High grade intraepithelial lesion, LSIL; Low grade intraepithelial lesion, CIN; Cervical intraepithelial neoplasia. ^a^ Samples that were HPV positive (GP5+/GP6+), but genotype was not identified.

Cytological diagnosis	HPV16 +	HPV18 +	Co infection	Unknown ^a^	HPV −	Total
*Invasive Cervical Cancer (ICC)*						
SCC	12	13	15	2	1	43
AC	-	-	1	-	-	1
ASC	3	-	-	-	-	3
*High Grade Neoplasia*						
HSIL (CIN II, CIN III)	2	2	6	-	1	11
*Low Grade Neoplasia*						
LSIL (CIN I)	-	-	3	1	-	4
Chronic cervicitis/metaplasia	2	4	7	-	2	15
Total	19	19	32	3	4	77

To determine the HPV status of the cervical cancer biopsies obtained for this study, DNA was extracted and HPV status determined by PCR with GP5+/GP6+ consensus primers that are specific for the L1 region of the HPV genome and used for the detection of a large spectrum of 44 genital HPV types [16]. In addition, type specific (TS) genotyping primers as described by Baay *et al.* (1996) were used to specifically detect HPV16 (TS 16) and HPV18 (TS 18) [17]. TS 16 anneals to the E6 coding region of HPV16 and TS 18 anneals to the L1 coding region of HPV18. These primer sets were included in our study because they have been shown to be more sensitive than the broad spectrum GP consensus primers [17]. In an effort to further increase the PCR detection sensitivity, a third primer set was included that specifically anneals to the E7 coding region of HPV16 (C16E7) and HPV18 (C18E7). 

**Figure 1:**
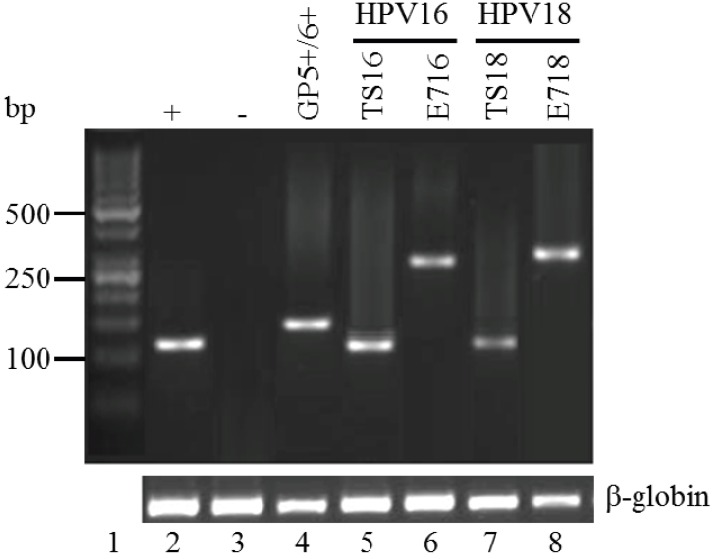
HPV detection by PCR. A representative image is shown. The molecular weight in base pairs (bp) is on the left (Lane 1). Lane 2: PCR reaction using TS18 primers and template DNA extracted from HeLa cells (positive control). Lane 3 is the negative control for the PCR reactions using template DNA extracted from a known HPV negative tumor biopsy with primer set GP5+/6+. All primer sets were tested with the negative control DNA alongside each PCR reaction performed (not shown). Lane 4 is the L1 amplicon produced by using GP5+/6+ primers in an HPV16/18 positive squamous cell carcinoma (SCC) lesion. Lanes 5 and 6 are the amplicons generated from HPV16 using TS16 and E716 primers, respectively, from DNA extracted from the same lesion as in Lane 4. This lesion was also positive with HPV18 specific primer sets (not shown). Lanes 7 and 8 are HPV18-specific amplicons produced with TS18 and E718 primers sets, respectively, from DNA extracted from an HPV18 positive SCC lesion. Amplified DNA bands were sequenced using the forward primer for each reaction to ensure amplification of type-specific HPV DNA.

To eliminate the possibility of false positive and false negative results. several controls were included in all PCR reactions; a known HPV negative paraffin-embedded tissue was incised on the microtome between test samples. DNA was extracted from this negative control sample and analyzed by PCR. As expected, this sample was negative for HPV DNA amplification, although amplification of the β-globin gene was possible, verifying DNA quality of this sample. DNA extracted from HeLa cells was used as positive control for HPV18 amplification (Figure 1). 

**Figure 2:**
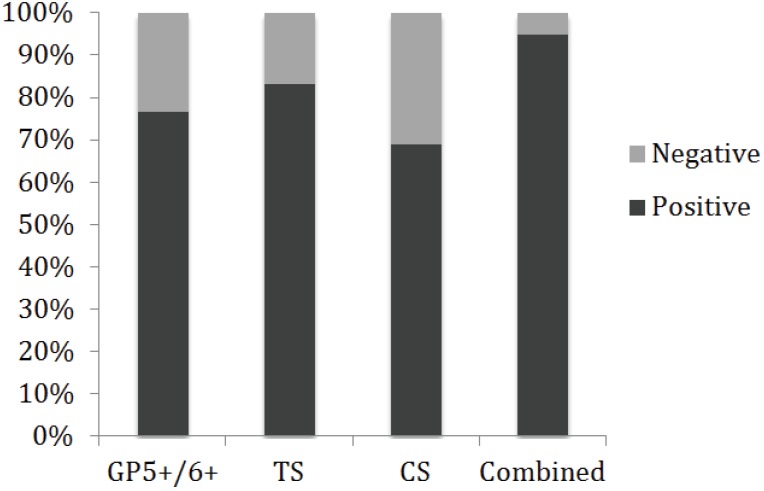
Detection of HPV in cervical lesions using alternative primer sets. Positive HPV detection using the broad range primer set GP5+/GP6+ was determined in 59 out of 77 samples. Sixty-four out of 77 samples were positive with TS primer sets and 53 out of 77 samples were positive with CS primer sets. In total, 73 out of 77 samples were positive with one or more primer sets, indicating an overall HPV prevalence of 94.81% in the cervical lesions tested.

Positive detection of the β-globin gene was achieved in all samples (data not shown). Positive detection of HPV DNA with the broad range detection primer set (GP) was achieved in 76.62% (59) of the samples (Figure 2), which is higher than the percentage of positive samples that has previously been achieved with the same primer set [17]. However, when the same samples were also tested with type specific primer pairs (TS16, TS18, C16E7 or C18E7), a total of 73 out of 77 (94.81%; 95% CI ±4.95) were found to be positive for HPV (Figure 2), which is consistent with the worldwide estimation of HPV infection in cervical carcinoma [19,20,21]. Only four of the biopsies were negative with all of the primer sets used in this study (5.19%). Of the 14 samples that were negative with the GP5+/GP6+ primers but positive with one or more of the type specific primer sets, 12 were positive with TS16 or TS18, six of which were also positive with either CS16 or CS18. Two samples were positive with our newly designed CS primer set but negative with all other primer sets used. Three samples were positive with the GP5+/GP6+ primer set that were negative with the type specific primer sets used, indicating that these samples were infected with an HPV type other than 16 or 18. 

In contrast to our results, a recent study conducted in Karachi, Pakistan concluded that HPV might not be the major cause of SCC of the cervix, as only 18% of SCC samples were found to be HPV positive [10]. However, the study by Yousuf *et al.* was limited to the use of GP5+/6+ consensus primers and a Seegene Assay (Seeplex Genotyping Kit). We and others have demonstrated that GP5+/6+ is less sensitve than other detection methods. In another study also using GP5+/6+ -mediated PCR detection followed by reverse-line blot hybridisation of the PCR products it was shown that the prevalence of HPV infection was low in the general population in Pakistan (2.8% in a cohort of healthy married women enrolled in a cervical screening program), but invasive cervical carcinoma (ICC) was primarily attributed to HPV infection (92.2%) [12]. Although these figures are in line with our data, the overall proportion of HPV positive carcinomas in our study is higher, presumably due to increased sensitivity with the range of PCR methods used. Importantly, a further study conducted in Karachi by Khan *et al.* (2007) [11] supports our data since a strong relationship between HPV infection and cervical cancer among Pakistani women was demonstrated: 98% of samples were HPV positive.

Analysis of samples with genotype specific primers revealed that HPV16 infection occurred either as a single infection or co-infection in 64.94% of samples, and HPV18 was detected in 66.23% of samples. Nineteen (24.68%; 95% CI ±9.63) of the samples were positive for HPV16 alone and 20 (25.97%; 95% CI 9.79) of the samples were positive for HPV18 alone. These data suggest the distribution of HPV16 and 18 in cervical lesions might be different from that in other regions of Pakistan, as in South Karachi the prevalence of HPV16 infection has been reported to be associated with 75%–93% of carcinomas whereas HPV18 infection is associated with 4%–6.6% [11,12]. Studies in other countries of a similar cultural background have also reported a high prevalence of HPV16 infection in cervical lesions collected from Iran (76% [22], 75% [23], 67.6% [24]) and Saudi Arabia (65.2% [25]) with a corresponding low HPV18 prevalence. However, another study conducted in Iran showed that HPV18 was more commonly detected in cervical lesions than HPV16 (41% HPV18 positive and 29.5% HPV16 positive) [26]. A high prevalence of HPV18 has also been reported in a study conducted in Indonesia with 44% of cervical cancer specimens testing positive for HPV16 and 39% positive for HPV18 [27]. 

**Figure 3:**
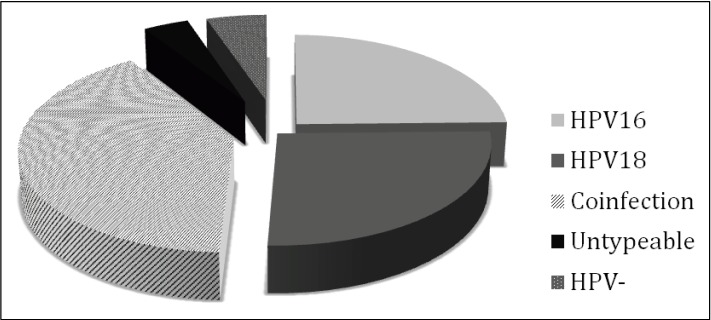
Distribution of HPV types 16 and 18 in cervical lesions. Seventy-three out of 77 samples tested were HPV positive, of which 31 were found co-infected with HPV16 and 18, 19 were positive for HPV 16 alone and 20 were positive for HPV18 alone. Three samples were HPV postive but the HPV type could not be determined.

Strikingly, 31 (40.26%; 95% CI ±10.95) samples in our study tested positive for both HPV16 and 18, indicating co-infection (Figure 3). This is significantly higher than the low incidence of HPV16/18 co-infection previously reported in Pakistan (<2%) [11,12] and in cervical cancers from Saudi Arabia (6.7%) and Jakarta, Indonesia (4.1%) [25,27]. However, a study in Thailand has indicated a high incidence of HPV16 (83.2%) and HPV18 (59.3%) in cervical cancer and lesions taken from women in four regions of Thailand and a high incidence of multiple infections, most notably 16/18 in 27.8% of samples and 16/18/11 in 20.4% of samples [28]. Co-infection with multiple HPV types was also noted in 15.3% of cervical specimens collected in Northwestern Iran but the majority of these cases of co-infection were HPV types 16 and 31 [24]. 

Why the incidence of HPV18 in Pakistan is so much higher in Punjab (this study) than in South Karachi [11,12] is not clear, but the more sensitive methods of detection used in our study could in part account for this. However, it is entirely possible that the prevalence of viral types differs between these regions of Pakistan. Whether this has any impact on the cervical cancer incidence and outcomes in the different regions remains to be determined. 

### 3.2. Histopathology, Age, and HPV Status

**Figure 4:**
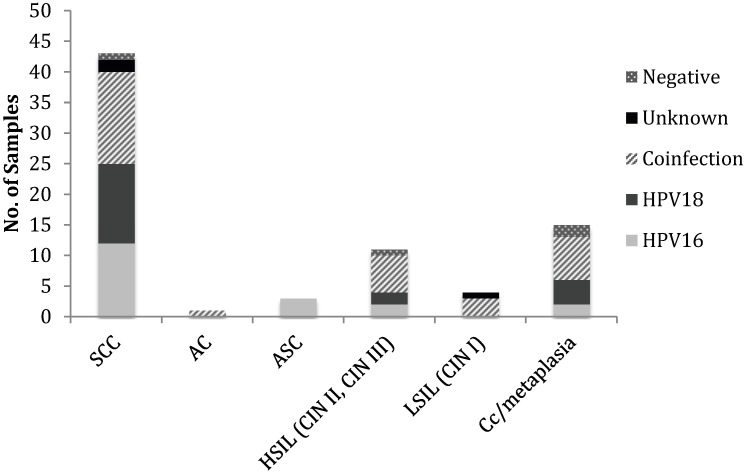
Genotypic distribution of HPV according to histopathological diagnosis. The incidence of SCC was higher and associated with HPV16, HPV18 and co-infection. The incidence of AC, ASC and LSIL were low. HSIL, chronic cervicitis (Cc)/metaplasia has a moderate incidence and were associated with higher incidence of co-infection in majority of cases as compared to HPV16 and 18 infection alone.

Correlation of histopathology with HPV status revealed that there was a high proportion of SCC (55.84% of samples, n = 43) that was equally associated with HPV16 (27.91%, n = 12) and 18 (30.23%, n = 13) alone (Table 3, Figure 4). Interestingly, a further 34.88% (n = 15) of these cases were associated with HPV16 and 18 co-infection. Two further SCC samples were positive with the GP5+/GP6+ primers but were not positive with any of the type specific primers, indicating that these samples were infected with an HPV type other than HPV16 or 18. One SCC case was negative with all the primer sets used for analysis, indicating that this tumor was HPV negative. AC was much less common (n = 1) and was only observed with co-infection of HPV16 and 18. ASC was also uncommon (n = 3) and was exclusive to HPV16 infection. Eleven of the samples were graded as HSIL, which was equally associated with HPV16 (n = 2) and 18 (n = 2) single infections. Six HSIL cases had a detectable HPV16 and 18 co-infection and one HSIL sample was negative with all primer sets, indicating that this sample was HPV negative. The number of LSIL samples in our cohort was low (n = 4) with the majority of these samples co-infected with HPV16 and 18 (n = 3). One of the LSIL samples was negative for HPV DNA in all of the assays used. There were 15 cases of chronic cervicitis and metaplasia, more than half of which were co-infected with HPV16 and 18 (n = 7) and two were negative with all primer sets used. 

**Figure 5:**
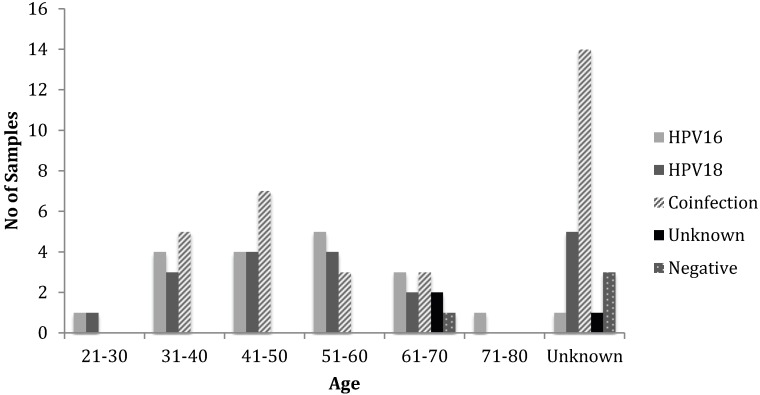
Genotypic distribution of HPV in cervical carcinoma samples in correlation with age. The maximum incidence of cervical carcinoma was found in age group 41–50 followed by the 31–40 and 51–60 age groups, respectively. The incidence of co-infection was also higher in age group 41–50, whereas, the incidences of HPV16 and 18 were higher in the 51–60 and 41–60 age groups, respectively.

Histopathological diagnosis was correlated with age and HPV status (Table 4, Figure 5). The overall age distribution for cancer incidence was 21 to 80. The age groups 21–30 and 71–80 only have few cases of cancer with the majority of cases in women aged between 31 and 70. Women aged between 41–50 had the highest incidence of cervical disease, which is consistent with the worldwide age distribution for cervical carcinoma [4]. 

**Table 4:** HPV16 and 18 prevalence in cervical cancer biopsies with age distribution.

Age Group	HPV16 +	HPV18 +	Co-infection	Genotype Unknown	HPV −	N
21–30	1	1	-	-	-	2
31–40	4	3	5	-	-	12
41–50	4	4	7	-	-	15
51–60	5	4	3	-	-	12
61–70	3	2	3	2	1	11
71–80	1	-	-	-	-	1
Unknown	1	5	14	1	3	24
**Total**	**19**	**19**	**32**	**3**	**4**	**77**

## 4. Conclusions

To date the prevalence and genotyping of HPV in cervical cancer patients in Southern Punjab of Pakistan has not been studied. In our study, 77 cervical samples were tested for HPV presence and 94.81% (n = 73) were found to be positive for HPV. The samples were also subjected to HPV genotype analysis by PCR with type specific primer sets; 19 samples (24.68%) were positive for HPV type 16 alone and 20 samples (25.97%) were positive for HPV type 18 alone. Thirty-one samples (40.26%) had a detectable co-infection with HPV16 and 18. This is the first study of its kind, which highlights the HPV prevalence in cervical cancers in this region. According to the International Association for Research in Cancer (IARC), the most prevalent high risk HPV genotypes which infect the cervix are: HPV16 (53%), HPV18 (15%), HPV45 (9%), HPV31 (6%), and HPV33 (3%) [29]. Reports on the prevalence of HPV genotypes in cervical cancer consistently show that HPV16 infection is the most common and that infection with HPV18 is the second most common [19,29]. Our results however demonstrate that the distribution of single HPV 16 and 18 infections in cervical lesions collected from Southern Pakistan does not follow this pattern; infection with HPV18 alone is as common as infection with HPV16 alone. Furthermore, the worldwide incidence of HPV16 and 18 co-infection is significantly lower than that detected in our samples [25,27,30,31]. While we acknowledge that the impact of our results would be enhanced by an increase in sample size and secondary validation of co-infection, such as Southern blot, at present neither of these options is available in the local setting. Nonetheless, with the measures we have taken to control for cross-contamination and non-specific amplification of related HPV species, we believe that our data are valid and provide insight into the prevalence of HPV16 and 18 in cervical lesions in a previously unstudied geographical region. 

The importance of high risk HPV infection in the development of cervical cancer has been clearly demonstrated [32]. Preventing the transmission of high risk HPV is difficult, therefore screening for persistent HPV infection and cytological analysis are the main defenses against cervical cancer and are widely practiced in the developed world [33]. In developed countries, routine screening by examination of Pap smears and cervical cytology are carried out. However, this method of screening is not entirely accurate for the detection of cancer and pre-cancerous lesions, requiring many rounds of screening to achieve programmatic effectiveness [34]. Since accurate virus typing plays an important role in clinical management of the disease [35], the Food and Drug Administration (FDA) has approved a PCR-based test, Cervista HPV HR, for screening of HPV infections in the general population. 

The general population and health care practitioners in Pakistan have little information and understanding about HPV infection, HPV associated cancers, their prevention, and HPV vaccination. Here, the only way to diagnose cancerous or pre-cancerous lesions is by means of Pap smear test, which as mentioned above is not altogether precise and accurate. In addition, Pap smears are not performed regularly and international recommendations are not adhered to. The inadequate diagnostic techniques, changing moral values, and lack of awareness are the main reasons for the current gradual increase in the incidence of disease in the country. 

Epidemiological evidence suggests that regular cervical screening in adult females is important in the reduction of HPV-associated cervical disease [36]. Cervical carcinoma screening tools currently used in Pakistan are limited to Pap smears in self referred patients to look for abnormal cellular changes followed by visual inspection of the cervix using acetic acid staining if abnormal cells are found in the initial Pap smear [37]. A routine screening program does not exist. Since a strong connection between HPV infection and the development of cervical cancer and neoplasia has been found in this study, HPV typing could be used as an effective test to predict malignant progression. Timely detection of a high risk HPV infection would allow effective follow-up and better management of disease, since early intervention has been shown to diminish the disease burden [38]. The surveillance of HPV infections and the impact of vaccination is a critical element in the process of HPV vaccine introduction. Based on this need, an HPV detection and genotyping test is likely to be made commercial very soon in the Atta-ur-Rahman School of Applied Biosciences, National University of Sciences and Technology (ASAB, NUST) diagnostic laboratory with a view to provide facility to risk prone women (diagnosed with abnormal Pap smears) so HPV infection status can be confirmed. 
